# The genome sequence of the Webb’s Wainscot, *Globia sparganii *(Esper, 1790)

**DOI:** 10.12688/wellcomeopenres.20181.1

**Published:** 2023-12-06

**Authors:** Gavin R. Broad, Natural History Museum Genome Acquisition Lab, Darwin Tree of Life Barcoding collective, Wellcome Sanger Institute Tree of Life programme, Wellcome Sanger Institute Scientific Operations: DNA Pipelines collective, Tree of Life Core Informatics collective, Darwin Tree of Life Consortium

**Affiliations:** 1 Natural History Museum, London, England, UK

**Keywords:** Globia sparganii, Webb’s Wainscot, genome sequence, chromosomal, Lepidoptera

## Abstract

We present a genome assembly from an individual male *Globia sparganii* (the Webb’s Wainscot; Arthropoda; Insecta; Lepidoptera; Noctuidae). The genome sequence is 676.7 megabases in span. Most of the assembly is scaffolded into 31 chromosomal pseudomolecules, including the Z sex chromosome. The mitochondrial genome has also been assembled and is 15.36 kilobases in length. Gene annotation of this assembly on Ensembl identified 18,385 protein coding genes.

## Species taxonomy

Eukaryota; Metazoa; Eumetazoa; Bilateria; Protostomia; Ecdysozoa; Panarthropoda; Arthropoda; Mandibulata; Pancrustacea; Hexapoda; Insecta; Dicondylia; Pterygota; Neoptera; Endopterygota; Amphiesmenoptera; Lepidoptera; Glossata; Neolepidoptera; Heteroneura; Ditrysia; Obtectomera; Noctuoidea; Noctuidae; Noctuinae; *Globia*; *Globia sparganii* (Esper, 1790) (NCBI:txid1660644).

## Background

*Globia sparganii*, Webb’s Wainscot, is one of many pale, buff-coloured noctuids called wainscots in English, not all particularly closely related (e.g., Davis *et al*., 2022). Webb’s Wainscot is relatively distinctive, with a small white kidney mark on the fore wing, partly enclosed by a black rim, part of a dark central streak down the wing. The extent of dark markings varies. Found across the Palaearctic, Webb’s Wainscot was formerly very localised in Britain, on the south coasts of England and Wales, but has been expanding its range across South-east England and East Anglia, with a more than 200% increase in range occupancy since the 1990s ( Randle *et al.*, 2019). The moth was named in English after Sydney Webb, who was the first person to find *G. sparganii* in Britain, in Kent in 1879 ( South, 1907), and Kent has always been a stronghold of this species. The Latin name refers to one of the foodplant genera, *Sparganium*, or Bur-reeds.

Moths are on the wing from July to October, and larvae feed in the stems of bulrushes and some other freshwater plants, such as Bur-reed and Yellow Flag Iris, in various freshwater bodies, from marshes to ponds and ditches ( Waring *et al.*, 2017). Adults often wander, and the first author has light-trapped them at home, far from any suitable habitat.

Along with some other stem-feeding noctuids, *Globia sparganii* has been sequenced in experiments to ascertain whether bracoviruses integrate into the genome, which they do; and whether that makes the species a potential non-target host for wasps used as biocontrol against the crop pest species, *Sesamia nonagrioides* (Lefèbvre), which it appears not to be ( Muller *et al.*, 2022).

## Genome sequence report

**Figure 1.**
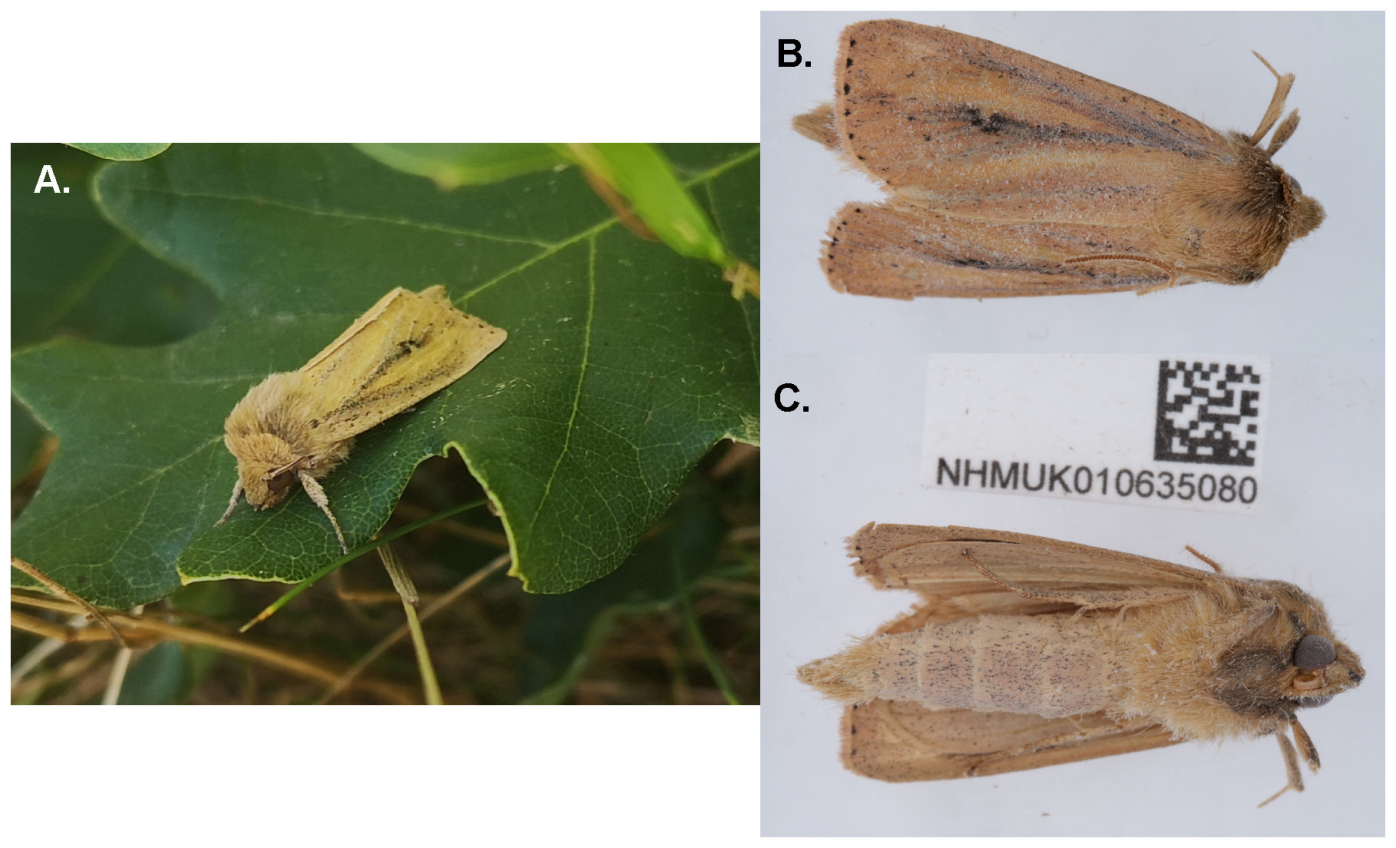
Photographs of the *Globia sparganii* (ilGloSpar1) specimen used for genome sequencing. **A**. Live specimen. **B**. Dorsal view and **C**. Ventral view of specimen during preservation and processing.

The genome was sequenced from one male *Globia sparganii* ( Figure 1) collected from Hever Castle, Kent, UK (51.19, 0.12). A total of 31-fold coverage in Pacific Biosciences single-molecule HiFi long reads was generated. Primary assembly contigs were scaffolded with chromosome conformation Hi-C data. Manual assembly curation corrected 11 missing joins or mis-joins and removed 6 haplotypic duplications, reducing the assembly length by 0.19% and the scaffold number by 3.92%.

**Figure 2.**
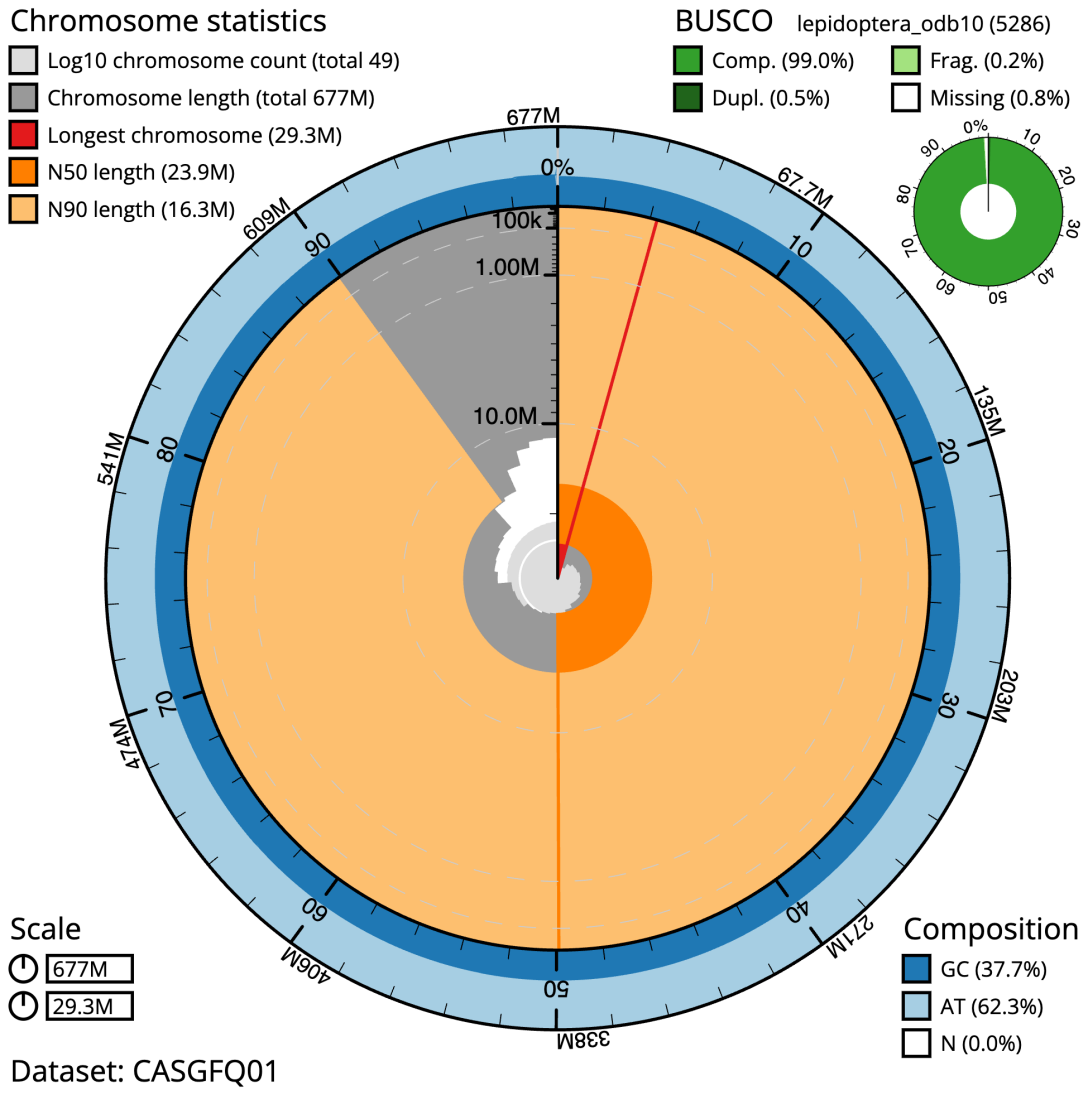
Genome assembly of *Globia sparganii*, ilGloSpar1.1: metrics. The BlobToolKit Snailplot shows N50 metrics and BUSCO gene completeness. The main plot is divided into 1,000 size-ordered bins around the circumference with each bin representing 0.1% of the 676,688,718 bp assembly. The distribution of scaffold lengths is shown in dark grey with the plot radius scaled to the longest scaffold present in the assembly (29,284,430 bp, shown in red). Orange and pale-orange arcs show the N50 and N90 scaffold lengths (23,857,693 and 16,301,409 bp), respectively. The pale grey spiral shows the cumulative scaffold count on a log scale with white scale lines showing successive orders of magnitude. The blue and pale-blue area around the outside of the plot shows the distribution of GC, AT and N percentages in the same bins as the inner plot. A summary of complete, fragmented, duplicated and missing BUSCO genes in the lepidoptera_odb10 set is shown in the top right. An interactive version of this figure is available at https://blobtoolkit.genomehubs.org/view/Globia%20sparganii/dataset/CASGFQ01/snail.

**Figure 3.**
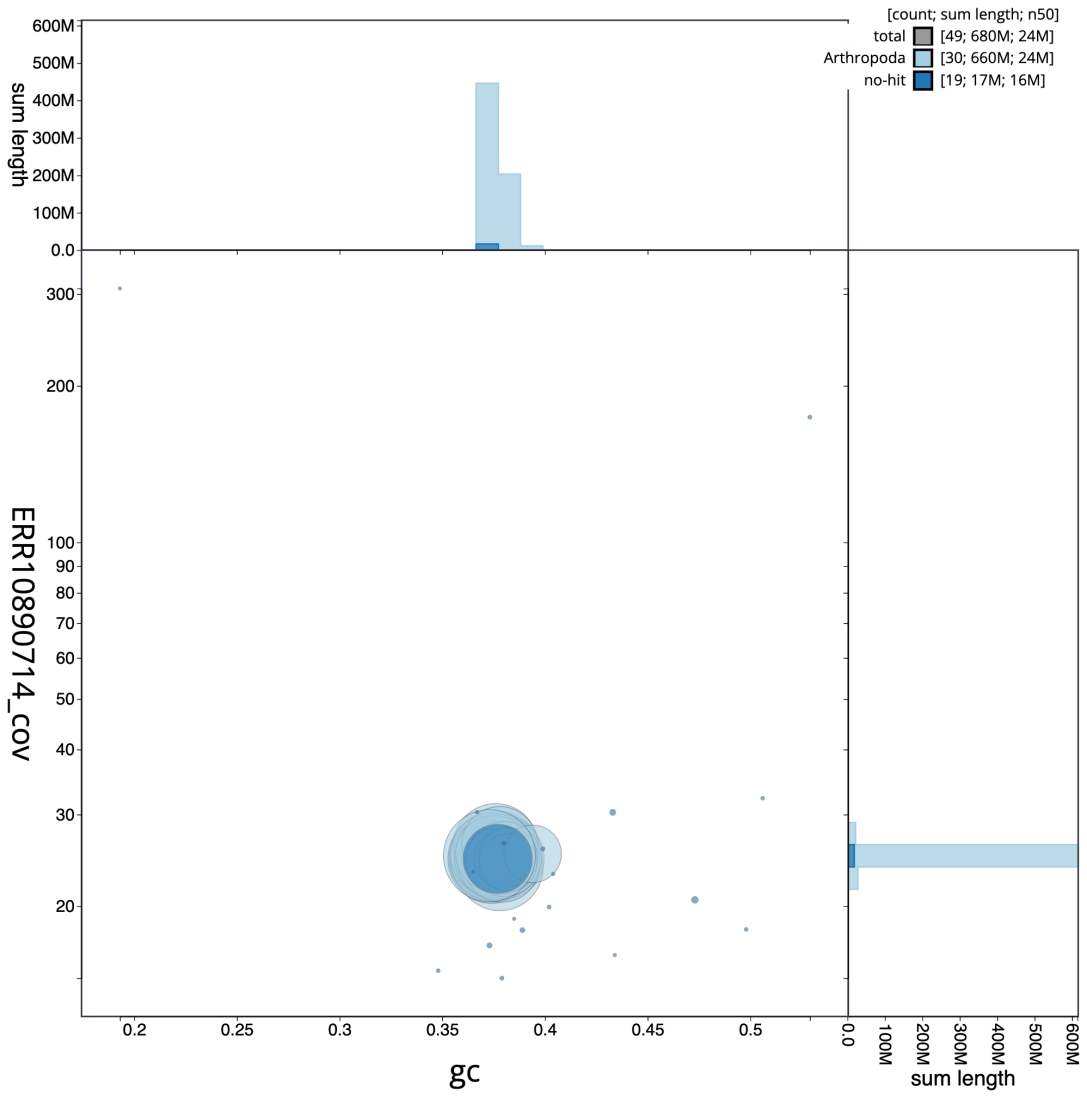
Genome assembly of *Globia sparganii*, ilGloSpar1.1: BlobToolKit GC-coverage plot. Scaffolds are coloured by phylum. Circles are sized in proportion to scaffold length. Histograms show the distribution of scaffold length sum along each axis. An interactive version of this figure is available at https://blobtoolkit.genomehubs.org/view/Globia%20sparganii/dataset/CASGFQ01/blob.

**Figure 4.**
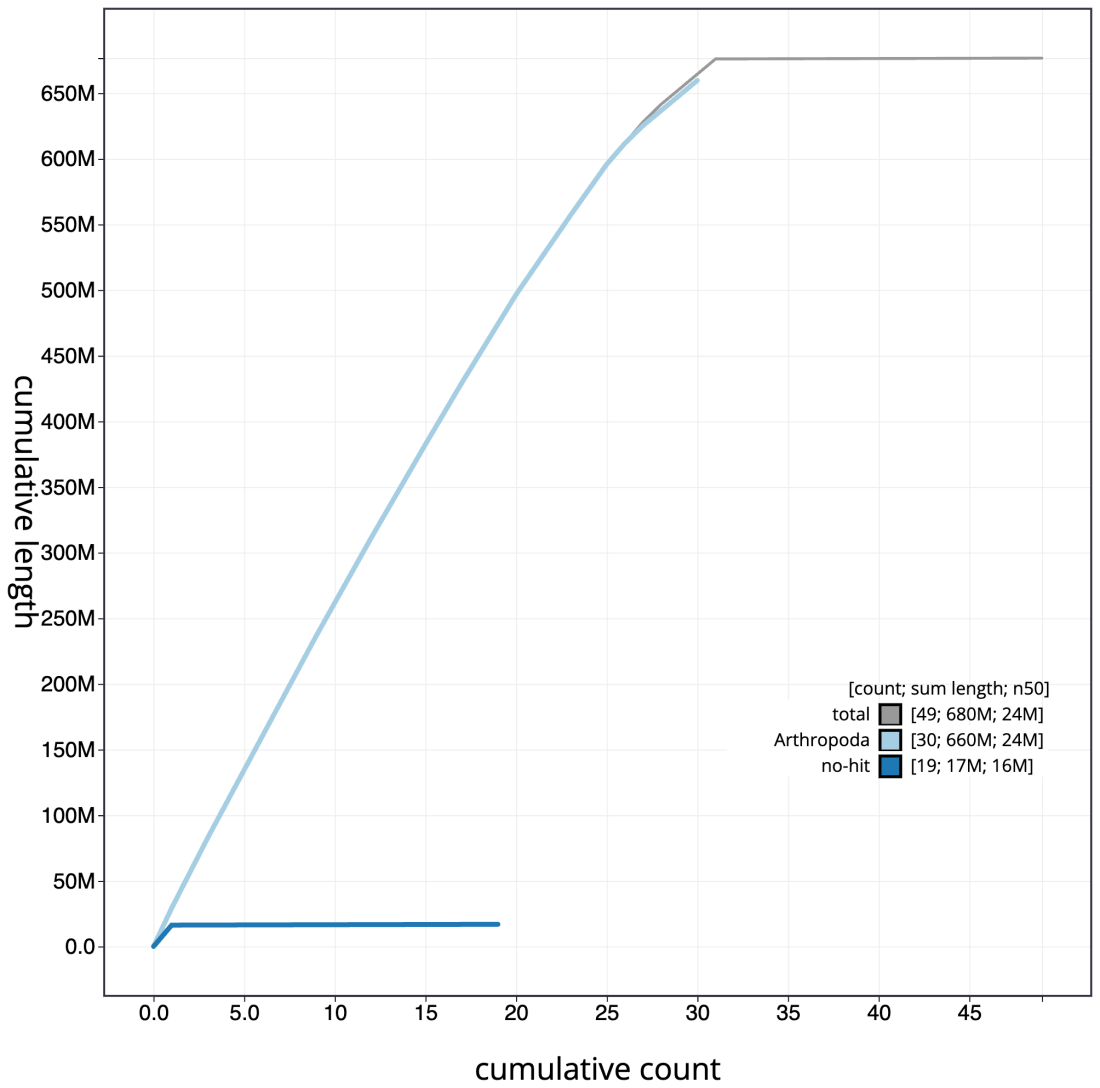
Genome assembly of *Globia sparganii*, ilGloSpar1.1: BlobToolKit cumulative sequence plot. The grey line shows cumulative length for all scaffolds. Coloured lines show cumulative lengths of scaffolds assigned to each phylum using the buscogenes taxrule. An interactive version of this figure is available at https://blobtoolkit.genomehubs.org/view/Globia%20sparganii/dataset/CASGFQ01/cumulative.

**Figure 5.**
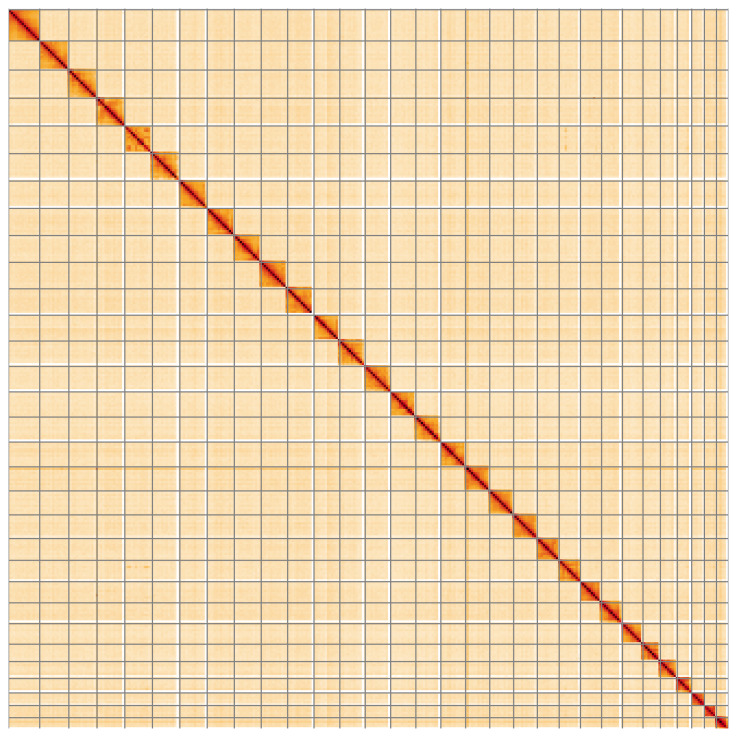
Genome assembly of *Globia sparganii*, ilGloSpar1.1: Hi-C contact map of the ilGloSpar1.1 assembly, visualised using HiGlass. Chromosomes are shown in order of size from left to right and top to bottom. An interactive version of this figure may be viewed at https://genome-note-higlass.tol.sanger.ac.uk/l/?d=G0o_g7xYQeOY77HCZpQi2A.

The final assembly has a total length of 676.7 Mb in 48 sequence scaffolds with a scaffold N50 of 23.9 Mb ( Table 1). The snailplot in Figure 2 provides a summary of the assembly statistics, while the distribution of assembly scaffolds on GC proportion and coverage is shown in Figure 3. The cumulative assembly plot in Figure 4 shows curves for subsets of scaffolds assigned to different phyla. Most (99.92%) of the assembly sequence was assigned to 31 chromosomal-level scaffolds, representing 30 autosomes and the Z sex chromosome. The Z chromosome was identified based on synteny with *Apamea epomidion* (GCA_947507525.1). Chromosome-scale scaffolds confirmed by the Hi-C data are named in order of size ( Figure 5; Table 2). While not fully phased, the assembly deposited is of one haplotype. Contigs corresponding to the second haplotype have also been deposited. The mitochondrial genome was also assembled and can be found as a contig within the multifasta file of the genome submission.

**Table 1.** Genome data for *Globia sparganii*, ilGloSpar1.1.

Project accession data		
Assembly identifier	ilGloSpar1.1	
Assembly release date	2023-03-10	
Species	*Globia sparganii*	
Specimen	ilGloSpar1	
NCBI taxonomy ID	1660644	
BioProject	PRJEB59770	
BioSample ID	SAMEA7849226	
Isolate information	ilGloSpar1	
Assembly metrics *		*Benchmark*
Consensus quality (QV)	65	*≥ 50*
*k*-mer completeness	100%	*≥ 95%*
BUSCO **	C:99.0%[S:98.6%,D:0.5%], F:0.2%,M:0.8%,n:5,286	*C ≥ 95%*
Percentage of assembly mapped to chromosomes	99.92%	*≥ 95%*
Sex chromosomes	Z chromosome	*localised homologous pairs*
Organelles	Mitochondrial genome assembled	*complete single alleles*
Raw data accessions		
PacificBiosciences SEQUEL II	ERR10879923, ERR10879922	
Hi-C Illumina	ERR10890717	
Genome assembly		
Assembly accession	GCA_949316385.1	
*Accession of alternate haplotype*	GCA_949316295.1	
Span (Mb)	676.7	
Number of contigs	135	
Contig N50 length (Mb)	11.4	
Number of scaffolds	48	
Scaffold N50 length (Mb)	23.9	
Longest scaffold (Mb)	29.3	
Genome annotation		
Number of protein-coding genes	18,385	
Number of gene transcripts	18,570	

* Assembly metric benchmarks are adapted from column VGP-2020 of “Table 1: Proposed standards and metrics for defining genome assembly quality” from ( Rhie *et al.*, 2021). ** BUSCO scores based on the lepidoptera_odb10 BUSCO set using v5.3.2. C = complete [S = single copy, D = duplicated], F = fragmented, M = missing, n = number of orthologues in comparison. A full set of BUSCO scores is available at https://blobtoolkit.genomehubs.org/view/Globia%20sparganii/dataset/CASGFQ01/busco.

**Table 2.** Chromosomal pseudomolecules in the genome assembly of *Globia sparganii*, ilGloSpar1.

INSDC accession	Chromosome	Length (Mb)	GC%
OX438653.1	1	27.33	37.5
OX438654.1	2	26.48	37.5
OX438655.1	3	26.07	37.5
OX438656.1	4	25.98	38.0
OX438657.1	5	25.71	37.5
OX438658.1	6	25.69	37.5
OX438659.1	7	25.47	37.5
OX438660.1	8	25.25	37.5
OX438661.1	9	24.81	37.5
OX438662.1	10	24.79	37.5
OX438663.1	11	24.26	37.5
OX438665.1	13	23.86	37.5
OX438664.1	12	23.86	37.5
OX438666.1	14	23.68	37.5
OX438667.1	15	23.45	37.5
OX438668.1	16	23.29	37.5
OX438669.1	17	22.61	38.0
OX438670.1	18	22.49	37.5
OX438671.1	19	22.29	38.0
OX438672.1	20	20.46	38.0
OX438673.1	21	19.99	38.0
OX438674.1	22	19.87	37.5
OX438675.1	23	19.55	38.0
OX438676.1	24	19.34	38.0
OX438677.1	25	16.3	37.5
OX438678.1	26	15.95	38.0
OX438679.1	27	13.44	38.0
OX438680.1	28	11.93	38.0
OX438681.1	29	11.35	38.0
OX438682.1	30	11.25	39.5
OX438652.1	Z	29.28	37.5
OX438683.1	MT	0.02	19.5

The estimated Quality Value (QV) of the final assembly is 65 with *k*-mer completeness of 100%, and the assembly has a BUSCO v5.3.2 completeness of 99.0% (single = 98.6%, duplicated = 0.5%), using the lepidoptera_odb10 reference set ( *n* = 5,286).

Metadata for specimens, barcode results, spectra estimates, sequencing runs, contaminants and pre-curation assembly statistics are given at https://links.tol.sanger.ac.uk/species/1660644.

## Genome annotation report

The *Globia sparganii* genome assembly (GCA_949316385.1) was annotated using the Ensembl rapid annotation pipeline ( Table 1; https://rapid.ensembl.org/Globia_sparganii_GCA_949316385.1/Info/Index). The resulting annotation includes 18,570 transcribed mRNAs from 18,385 protein-coding genes.

## Methods

### Sample acquisition and nucleic acid extraction

A male *Globia sparganii* (specimen ID NHMUK010635080, ToLID ilGloSpar1) was collected from Hever Castle, Hever, Kent, UK (latitude 51.19, longitude 0.12) on 2020-08-27 using a light trap. The specimen was collected and identified by Gavin Broad (Natural History Museum) and preserved on dry ice.

DNA was extracted at the Tree of Life laboratory, Wellcome Sanger Institute (WSI). The ilGloSpar1 sample was weighed and dissected on dry ice with tissue set aside for Hi-C sequencing. Abdomen tissue was cryogenically disrupted to a fine powder using a Covaris cryoPREP Automated Dry Pulveriser, receiving multiple impacts. High molecular weight (HMW) DNA was extracted using the Qiagen MagAttract HMW DNA extraction kit. HMW DNA was sheared into an average fragment size of 12–20 kb in a Megaruptor 3 system with speed setting 30. Sheared DNA was purified by solid-phase reversible immobilisation using AMPure PB beads with a 1.8X ratio of beads to sample to remove the shorter fragments and concentrate the DNA sample. The concentration of the sheared and purified DNA was assessed using a Nanodrop spectrophotometer and Qubit Fluorometer and Qubit dsDNA High Sensitivity Assay kit. Fragment size distribution was evaluated by running the sample on the FemtoPulse system.

### Sequencing

Pacific Biosciences HiFi circular consensus DNA sequencing libraries were constructed according to the manufacturers’ instructions. DNA and RNA sequencing was performed by the Scientific Operations core at the WSI on a Pacific Biosciences SEQUEL II (HiFi) instrument. Hi-C data were also generated from head and thorax tissue of ilGloSpar1 using the Arima2 kit and sequenced on the Illumina NovaSeq 6000 instrument.

### Genome assembly, curation and evaluation

Assembly was carried out with Hifiasm ( Cheng *et al.*, 2021) and haplotypic duplication was identified and removed with purge_dups ( Guan *et al.*, 2020). The assembly was then scaffolded with Hi-C data ( Rao *et al.*, 2014) using YaHS ( Zhou *et al.*, 2023). The assembly was checked for contamination and corrected as described previously ( Howe *et al.*, 2021). Manual curation was performed using HiGlass ( Kerpedjiev *et al.*, 2018) and Pretext ( Harry, 2022). The mitochondrial genome was assembled using MitoHiFi ( Uliano-Silva *et al.*, 2023), which runs MitoFinder ( Allio *et al.*, 2020) or MITOS ( Bernt *et al.*, 2013) and uses these annotations to select the final mitochondrial contig and to ensure the general quality of the sequence.

A Hi-C map for the final assembly was produced using bwa-mem2 ( Vasimuddin *et al.*, 2019) in the Cooler file format ( Abdennur & Mirny, 2020). To assess the assembly metrics, the *k*-mer completeness and QV consensus quality values were calculated in Merqury ( Rhie *et al.*, 2020). This work was done using Nextflow ( Di Tommaso *et al.*, 2017) DSL2 pipelines “sanger-tol/readmapping” ( Surana *et al.*, 2023a) and “sanger-tol/genomenote” ( Surana *et al.*, 2023b). The genome was analysed within the BlobToolKit environment ( Challis *et al.*, 2020) and BUSCO scores ( Manni *et al.*, 2021; Simão *et al.*, 2015) were calculated.

Table 3 contains a list of relevant software tool versions and sources.

**Table 3.** Software tools: versions and sources.

Software tool	Version	Source
BlobToolKit	4.1.7	https://github.com/blobtoolkit/blobtoolkit
BUSCO	5.3.2	https://gitlab.com/ezlab/busco
Hifiasm	0.16.1-r375	https://github.com/chhylp123/hifiasm
HiGlass	1.11.6	https://github.com/higlass/higlass
Merqury	MerquryFK	https://github.com/thegenemyers/MERQURY.FK
MitoHiFi	2	https://github.com/marcelauliano/MitoHiFi
PretextView	0.2	https://github.com/wtsi-hpag/PretextView
purge_dups	1.2.3	https://github.com/dfguan/purge_dups
sanger-tol/genomenote	v1.0	https://github.com/sanger-tol/genomenote
sanger-tol/readmapping	1.1.0	https://github.com/sanger-tol/readmapping/tree/1.1.0
YaHS	1.2a	https://github.com/c-zhou/yahs

### Genome annotation

The BRAKER2 pipeline ( Brůna *et al.*, 2021) was used in the default protein mode to generate annotation for the *Globia sparganii* assembly (GCA_949316385.1) in Ensembl Rapid Release.

### Wellcome Sanger Institute – Legal and Governance

The materials that have contributed to this genome note have been supplied by a Darwin Tree of Life Partner. The submission of materials by a Darwin Tree of Life Partner is subject to the **‘Darwin Tree of Life Project Sampling Code of Practice’**, which can be found in full on the Darwin Tree of Life website here. By agreeing with and signing up to the Sampling Code of Practice, the Darwin Tree of Life Partner agrees they will meet the legal and ethical requirements and standards set out within this document in respect of all samples acquired for, and supplied to, the Darwin Tree of Life Project. 

Further, the Wellcome Sanger Institute employs a process whereby due diligence is carried out proportionate to the nature of the materials themselves, and the circumstances under which they have been/are to be collected and provided for use. The purpose of this is to address and mitigate any potential legal and/or ethical implications of receipt and use of the materials as part of the research project, and to ensure that in doing so we align with best practice wherever possible. The overarching areas of consideration are:

• Ethical review of provenance and sourcing of the material

• Legality of collection, transfer and use (national and international) 

Each transfer of samples is further undertaken according to a Research Collaboration Agreement or Material Transfer Agreement entered into by the Darwin Tree of Life Partner, Genome Research Limited (operating as the Wellcome Sanger Institute), and in some circumstances other Darwin Tree of Life collaborators.

## Data Availability

European Nucleotide Archive: *Globia sparganii* (Webb's wainscot). Accession number PRJEB59770; https://identifiers.org/ena.embl/PRJEB59770. ( Wellcome Sanger Institute, 2023) The genome sequence is released openly for reuse. The *Globia sparganii* genome sequencing initiative is part of the Darwin Tree of Life (DToL) project. All raw sequence data and the assembly have been deposited in INSDC databases. Raw data and assembly accession identifiers are reported in Table 1.

## Author information

Members of the Natural History Museum Genome Acquisition Lab are listed here: https://doi.org/10.5281/zenodo.4790042.

Members of the Darwin Tree of Life Barcoding collective are listed here: https://doi.org/10.5281/zenodo.4893703.

Members of the Wellcome Sanger Institute Tree of Life programme are listed here: https://doi.org/10.5281/zenodo.4783585.

Members of Wellcome Sanger Institute Scientific Operations: DNA Pipelines collective are listed here: https://doi.org/10.5281/zenodo.4790455.

Members of the Tree of Life Core Informatics collective are listed here: https://doi.org/10.5281/zenodo.5013541.

Members of the Darwin Tree of Life Consortium are listed here: https://doi.org/10.5281/zenodo.4783558.

## References

[ref-1] Abdennur N Mirny LA : Cooler: Scalable storage for Hi-C data and other genomically labeled arrays. *Bioinformatics.* 2020;36(1):311–316. 10.1093/bioinformatics/btz540 31290943 PMC8205516

[ref-2] Allio R Schomaker‐Bastos A Romiguier J : MitoFinder: Efficient automated large‐scale extraction of mitogenomic data in target enrichment phylogenomics. *Mol Ecol Resour.* 2020;20(4):892–905. 10.1111/1755-0998.13160 32243090 PMC7497042

[ref-4] Bernt M Donath A Jühling F : MITOS: Improved *de novo* metazoan mitochondrial genome annotation. *Mol Phylogenet Evol.* 2013;69(2):313–319. 10.1016/j.ympev.2012.08.023 22982435

[ref-29] Brůna T Hoff KJ Lomsadze A : BRAKER2: Automatic eukaryotic genome annotation with GeneMark-EP+ and AUGUSTUS supported by a protein database. *NAR Genom Bioinform.* 2021;3(1): lqaa108. 10.1093/nargab/lqaa108 33575650 PMC7787252

[ref-5] Challis R Richards E Rajan J : BlobToolKit - interactive quality assessment of genome assemblies. *G3 (Bethesda).* 2020;10(4):1361–1374. 10.1534/g3.119.400908 32071071 PMC7144090

[ref-7] Cheng H Concepcion GT Feng X : Haplotype-resolved *de novo* assembly using phased assembly graphs with hifiasm. *Nat Methods.* 2021;18(2):170–175. 10.1038/s41592-020-01056-5 33526886 PMC7961889

[ref-30] Davis RB Õunap E Tammaru T : A supertree of Northern European macromoths. *PLoS One.* 2022;17(2): e0264211. 10.1371/journal.pone.0264211 35180261 PMC8856531

[ref-8] Di Tommaso P Chatzou M Floden EW : Nextflow enables reproducible computational workflows. *Nat Biotechnol.* 2017;35(4):316–319. 10.1038/nbt.3820 28398311

[ref-10] Guan D McCarthy SA Wood J : Identifying and removing haplotypic duplication in primary genome assemblies. *Bioinformatics.* 2020;36(9):2896–2898. 10.1093/bioinformatics/btaa025 31971576 PMC7203741

[ref-12] Harry E : PretextView (Paired REad TEXTure Viewer): A desktop application for viewing pretext contact maps. 2022; [Accessed 19 October 2022]. Reference Source

[ref-13] Howe K Chow W Collins J : Significantly improving the quality of genome assemblies through curation. *GigaScience.* Oxford University Press,2021;10(1): giaa153. 10.1093/gigascience/giaa153 33420778 PMC7794651

[ref-14] Kerpedjiev P Abdennur N Lekschas F : HiGlass: web-based visual exploration and analysis of genome interaction maps. *Genome Biol.* 2018;19(1): 125. 10.1186/s13059-018-1486-1 30143029 PMC6109259

[ref-16] Manni M Berkeley MR Seppey M : BUSCO update: Novel and streamlined workflows along with broader and deeper phylogenetic coverage for scoring of eukaryotic, prokaryotic, and viral genomes. *Mol Biol Evol.* 2021;38(10):4647–4654. 10.1093/molbev/msab199 34320186 PMC8476166

[ref-31] Muller H Heisserer C Fortuna T : Investigating bracovirus chromosomal integration and inheritance in lepidopteran host and nontarget species. *Mol Ecol.* 2022;31(21):5538–5551. 10.1111/mec.16685 36070218

[ref-32] Randle Z Evans-Hill LJ Parsons MS : Atlas of Britain & Ireland’s Larger Moths. Newbury: NatureBureau,2019. Reference Source

[ref-17] Rao SSP Huntley MH Durand NC : A 3D map of the human genome at kilobase resolution reveals principles of chromatin looping. *Cell.* 2014;159(7):1665–1680. 10.1016/j.cell.2014.11.021 25497547 PMC5635824

[ref-18] Rhie A McCarthy SA Fedrigo O : Towards complete and error-free genome assemblies of all vertebrate species. *Nature.* 2021;592(7856):737–746. 10.1038/s41586-021-03451-0 33911273 PMC8081667

[ref-19] Rhie A Walenz BP Koren S : Merqury: Reference-free quality, completeness, and phasing assessment for genome assemblies. *Genome Biol.* 2020;21(1): 245. 10.1186/s13059-020-02134-9 32928274 PMC7488777

[ref-20] Simão FA Waterhouse RM Ioannidis P : BUSCO: assessing genome assembly and annotation completeness with single-copy orthologs. *Bioinformatics.* 2015;31(19):3210–3212. 10.1093/bioinformatics/btv351 26059717

[ref-33] South R : The Moths of the British Isles. London: Frederick Warne & Co,1907. Reference Source

[ref-22] Surana P Muffato M Qi G : sanger-tol/readmapping: sanger-tol/readmapping v1.1.0 - Hebridean Black (1.1.0). *Zenodo.* 2023a; [Accessed 21 July 2023]. 10.5281/zenodo.7755665

[ref-23] Surana P Muffato M Sadasivan Baby C : sanger-tol/genomenote (v1.0.dev). *Zenodo.* 2023b; [Accessed 21 July 2023]. 10.5281/zenodo.6785935

[ref-24] Uliano-Silva M Ferreira JGRN Krasheninnikova K : MitoHiFi: a python pipeline for mitochondrial genome assembly from PacBio high fidelity reads. *BMC Bioinformatics.* 2023;24(1): 288. 10.1186/s12859-023-05385-y 37464285 PMC10354987

[ref-25] Vasimuddin M Misra S Li H : Efficient Architecture-Aware Acceleration of BWA-MEM for Multicore Systems.In: *2019 IEEE International Parallel and Distributed Processing Symposium (IPDPS).*IEEE,2019;314–324. 10.1109/IPDPS.2019.00041

[ref-34] Waring P Townsend M Lewington R : Field Guide to the Moths of Great Britain and Ireland: Third Edition. Bloomsbury Wildlife Guides,2017. Reference Source

[ref-28] Wellcome Sanger Institute: The genome sequence of the Webb’s Wainscot, *Globia sparganii* (Esper, 1790). European Nucleotide Archive.[dataset], accession number PRJEB59770,2023. 10.12688/wellcomeopenres.20181.1 PMC11480736 39417200

[ref-27] Zhou C McCarthy SA Durbin R : YaHS: yet another Hi-C scaffolding tool. *Bioinformatics.* 2023;39(1): btac808. 10.1093/bioinformatics/btac808 36525368 PMC9848053

