# Supplementary material for: The genome sequence of the Webb’s Wainscot, Globia sparganii (Esper, 1790)
Source: Wellcome Open Res. 2023 Dec 6;8:565. [Version 1] doi: 10.12688/wellcomeopenres.20181.1 (PMC11480736; doi:10.12688/wellcomeopenres.20181.1)
Supplement: Peer review history

# Reviewer report: Reviewer response for version 1

**DOI:** 10.21956/wellcomeopenres.22344.r102150

**Published:** 2024-10-15

**Authors:** Panagiotis Ioannidis

**Affiliations:** 1 Foundation for Research & Technology - Hellas, Heraklion, Crete, GR 70013, Greece

This genome report describes the sequencing and annotation of the genome of the lepidopteran *Globia sparganii*. The methodology is well-described with enough details so that the reader can understand how exactly every analysis was performed. I particularly like the fact that the authors have also done genome annotation and they provided a gene set via the Ensembl rapid pipeline.

**I have a few minor comments:**

 1) the Introduction needs some editing since some expressions sound a bit odd. For example, the beginning of the second paragraph reads "Moths are on the wing" which I presume means that they are in their adult stage. In the same paragraph the authors say "...the first author has light-trapped them at home..." which I believe that can be rephrased to something more "formal". Finally, I cannot understand the meaning of the last paragraph. I believe that the authors try to say why this species is important, but they don't clearly say it! Instead, the written text sounds more like something only the experts would know (about bracoviruses and how their genomic integration might affect their biocontrol)... I would also rephrase this paragraph to something simpler and clearer.

 2) In the Methods section, in the "Sequencing" sub-section the authors say that "DNA and RNA sequencing was performed...", but nowhere else in the manuscript they refer to RNAseq data. Also, I couldn't see any deposited RNAseq data in the BioProject. So, I guess that they wrote "RNA" by mistake and they should delete it from the sentence.

 3) As I said earlier, I noticed that the authors also provide a gene set for this species. In such cases, it is a very nice idea (an absolute "must" in my opinion) to include BUSCO scores for the predicted gene sets as well. This way, the reader can compare the BUSCO score of the genome assembly to the BUSCO score of the gene set predicted on this genome assembly. Such a comparison tells you how good the gene prediction pipeline is, and if it can be improved (i.e. in the case where considerably less genes are found in the genome assembly than in the predicted gene set).

Are sufficient details of methods and materials provided to allow replication by others?

Yes

Is the rationale for creating the dataset(s) clearly described?

Yes

Are the datasets clearly presented in a useable and accessible format?

Yes

Are the protocols appropriate and is the work technically sound?

Yes

Reviewer Expertise:

Insect genomics

I confirm that I have read this submission and believe that I have an appropriate level of expertise to confirm that it is of an acceptable scientific standard.

# Reviewer report: Reviewer response for version 1

**DOI:** 10.21956/wellcomeopenres.22344.r102144

**Published:** 2024-10-07

**Authors:** Sophie Tandonnet

**Affiliations:** 1 University of Barcelona, Barcelona, Spain

In this genome note, the authors report the chromosomal-scale assembly of the genome of Globia sparganii (676.7 Mb). The assembly is highly continuous and complete (99.0% complete BUSCOs) and originates from only one individual male (specimen ilGloSpar1). 

 The assembly is based on Pacbio HiFi long reads and scaffolded into pseudo-chromosomal molecules using Hi-C data. The assembly has a corresponding annotation, which I could download without problem. This annotation includes 18,385 protein coding genes. 

 All in all this note reports a new, high quality genome assembly along with its corresponding annotation. 

 My main suggestion is to add more details (see points below), especially about RNA sequencing, genome annotation, and sex chromosome identification.

  

- The text mentions "The Z chromosome was identified based on synteny with *Apamea epomidion* (GCA_947507525.1).": It is not clear to me how the Z chromosome was identified in A. epomidion. It seems it was also identified based on synteny. This chain of synteny analyses to identify the sex chromosomes is a bit tenuous. I suggest the authors rephrase saying "the putative Z chromosome…". I also suggest that the synteny analysis is explained in greater details (programs used, species…) and, if it exists, to add a reference about the conservation of macrosynteny in Noctuidae.
- The text mentions RNA sequencing was performed: I couldn't find this data although maybe it is in this page: https://www.ebi.ac.uk/ena/browser/view/PRJEB59769 (RNA sequencing is also mentioned)?  If it exists, I suggest referring to it (adding the accessions numbers) and adding the details of how it was extracted and sequenced, as well as adding the specimen details (sex, developmental stage, tissue, etc). This data should also be used for the genome annotation. 
- In the background it is mentioned that "bracoviruses integrate into the genome". Was the localization of such bracoviruses identified? 
- The genome annotation section is missing details: were the repeats annotated? Which database of proteins was used in Braker2?
- Were any symbionts or co-bionts found ?

Are sufficient details of methods and materials provided to allow replication by others?

Partly

Is the rationale for creating the dataset(s) clearly described?

Yes

Are the datasets clearly presented in a useable and accessible format?

Yes

Are the protocols appropriate and is the work technically sound?

Yes

Reviewer Expertise:

Evolutionary biology, genomics, non-model organisms

I confirm that I have read this submission and believe that I have an appropriate level of expertise to confirm that it is of an acceptable scientific standard.

# Reviewer report: Reviewer response for version 1

**DOI:** 10.21956/wellcomeopenres.22344.r84708

**Published:** 2024-08-26

**Authors:** Galarza Juan

**Affiliations:** 1 University of Oulu, Oulu, Finland

In this paper, the genome of Globia sparganii, also known as Webb's Wainscot, has been assembled from a male specimen collected in Kent, UK. This noctuid moth's genome spans 676.7 megabases and is primarily scaffolded into 31 chromosomal pseudomolecules, including the Z sex chromosome. The mitochondrial genome is also included, spanning 15.36 kilobases. The genome annotation identified 18,385 protein-coding genes. The moth's larvae feed on freshwater plants, and recent studies have sequenced its genome to understand bracovirus integration, finding no risk as a non-target host for biocontrol wasps.

 While the text provides a high-level overview of the methods, it does not specify some of the details that may be required for complete replication, such as the specific enzymes or software version used. However, a researcher with experience in genome sequencing and annotation should be able to replicate the methods described in the text with a high degree of accuracy. Overall, the core data (genome assembly and likely annotations) seem accessible, although I didn’t verify all the links. Finally, the text describes the moth Globia sparganii but doesn't explicitly mention why the researchers created a dataset related to it.

Are sufficient details of methods and materials provided to allow replication by others?

Partly

Is the rationale for creating the dataset(s) clearly described?

Partly

Are the datasets clearly presented in a useable and accessible format?

Yes

Are the protocols appropriate and is the work technically sound?

Yes

Reviewer Expertise:

Bioinformatics

I confirm that I have read this submission and believe that I have an appropriate level of expertise to confirm that it is of an acceptable scientific standard, however I have significant reservations, as outlined above.
